# Photo Quiz: Hematuria in a pediatric patient

**DOI:** 10.1128/jcm.00241-24

**Published:** 2025-03-12

**Authors:** Víctor Antón Berenguer, Sara Ruiz González, Francisco Jesús Merino Fernández, José Miguel Rubio Muñoz, Sara Gómez de Frutos, María Delmans Flores-Chavez

**Affiliations:** 1 Microbiology and Parasitology Department, Severo Ochoa University Hospital 16857 , Madrid, Spain; 2 Paediatrics Department, Severo Ochoa University Hospital 16857 , Madrid, Spain; 3 Reference and Research Laboratory for Parasitology, National Microbiology Centre, Instituto de Salud Carlos III 38176 , Madrid, Spain; 4 Biomedical Research Networking Centre of Infectious Diseases (CIBERINFEC), Instituto de Salud Carlos III 38176 , Madrid, Spain; 5 Fundación Mundo Sano 681353 , Madrid, Spain; Mayo Clinic Minnesota, Rochester, Minnesota, USA

## PHOTO QUIZ 

A 12-year-old male was admitted to the emergency department of a secondary hospital in the south of Madrid community (Spain) with 1 week of terminal hematuria accompanied by abdominal colic-like pain with urination. The patient presented with neither fever nor genitourinary alterations and denied both the intake of any food capable of dyeing urine or swimming in rivers or lakes. He emigrated with his family from Gambia a year ago, with no recent travels to their original country and no additional personal or familiar clinically relevant history findings.

A urine specimen showed macroscopic hematuria with no apparent clots. Urine sediment microscopy confirmed hematuria (25–30 erythrocytes/field), a slight leukocyturia (6–10 leukocytes/field), and proteinuria (protein/creatinine index 0.3 mg/mg).

Blood count and renal function biochemistry tests in serum (creatinine, urea, uric acid, and ions) were not revealing, with no significant abnormalities in eosinophil percentage (7.5%; normal values 0%–7%) or in eosinophil concentration (0.43 × 10^3^ eosinophils/µL; normal values 0–0.8 × 10^3^ eosinophils/µL). The patient was discharged with empiric oral fosfomycin/trometamol after collecting an additional urine specimen for culture and ova and parasite study. Two days later, the patient returned to the emergency department due to the persistence of abdominal pain and hematuria with no improvement on antibiotic therapy. The patient also underwent an abdominal echography, revealing a thickening of the left anterolateral bladder wall.

**Fig 1:**
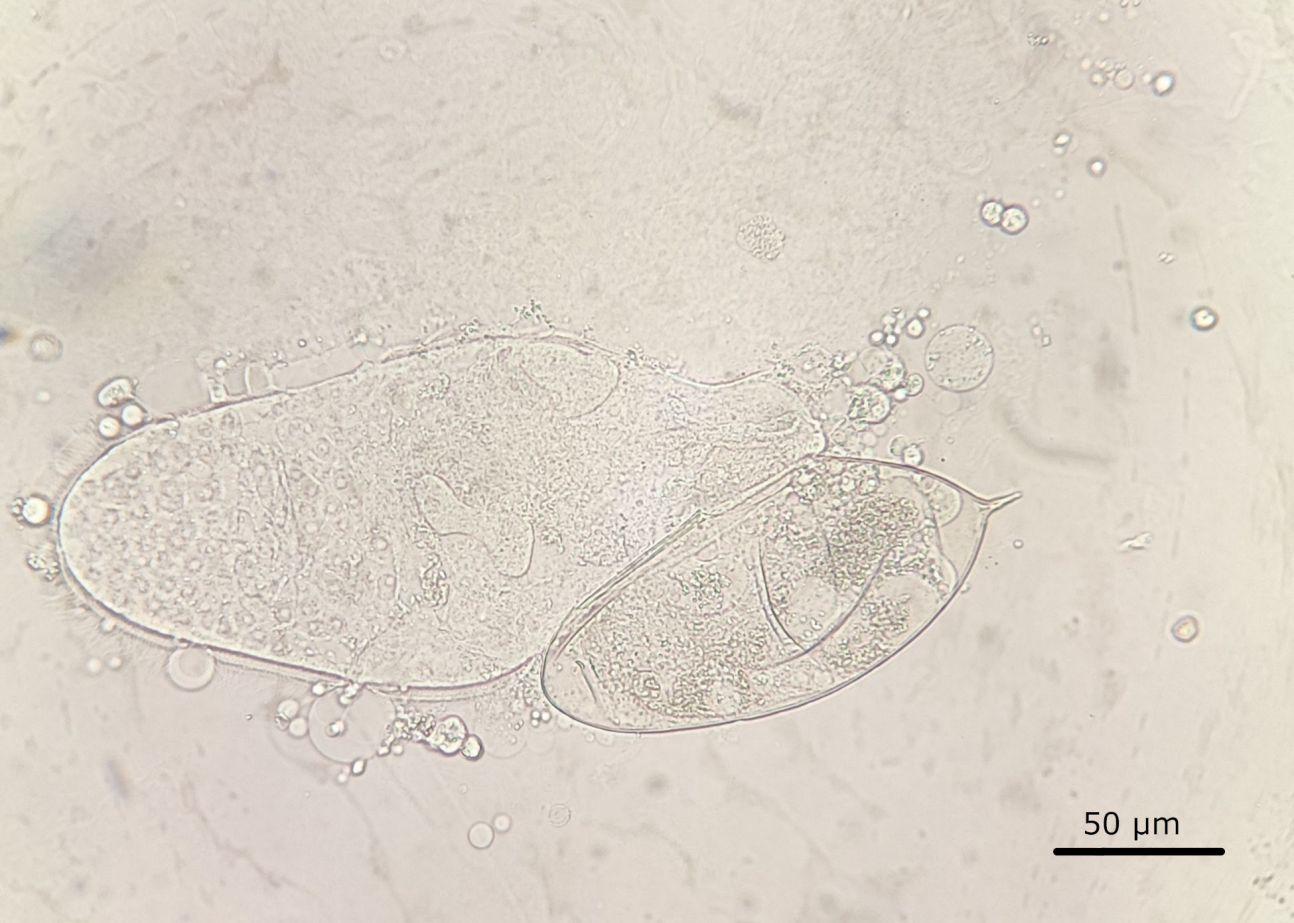
Microscopic image of urine specimen after centrifugation (1,500 rpm for 10 minutes), without staining (magnification, 400×).

There was no growth from the urine culture. After specimen concentration by centrifugation, microscopic examination revealed objects as those represented in Fig. 1.

## ANSWER TO PHOTO QUIZ

**Fig 2:**
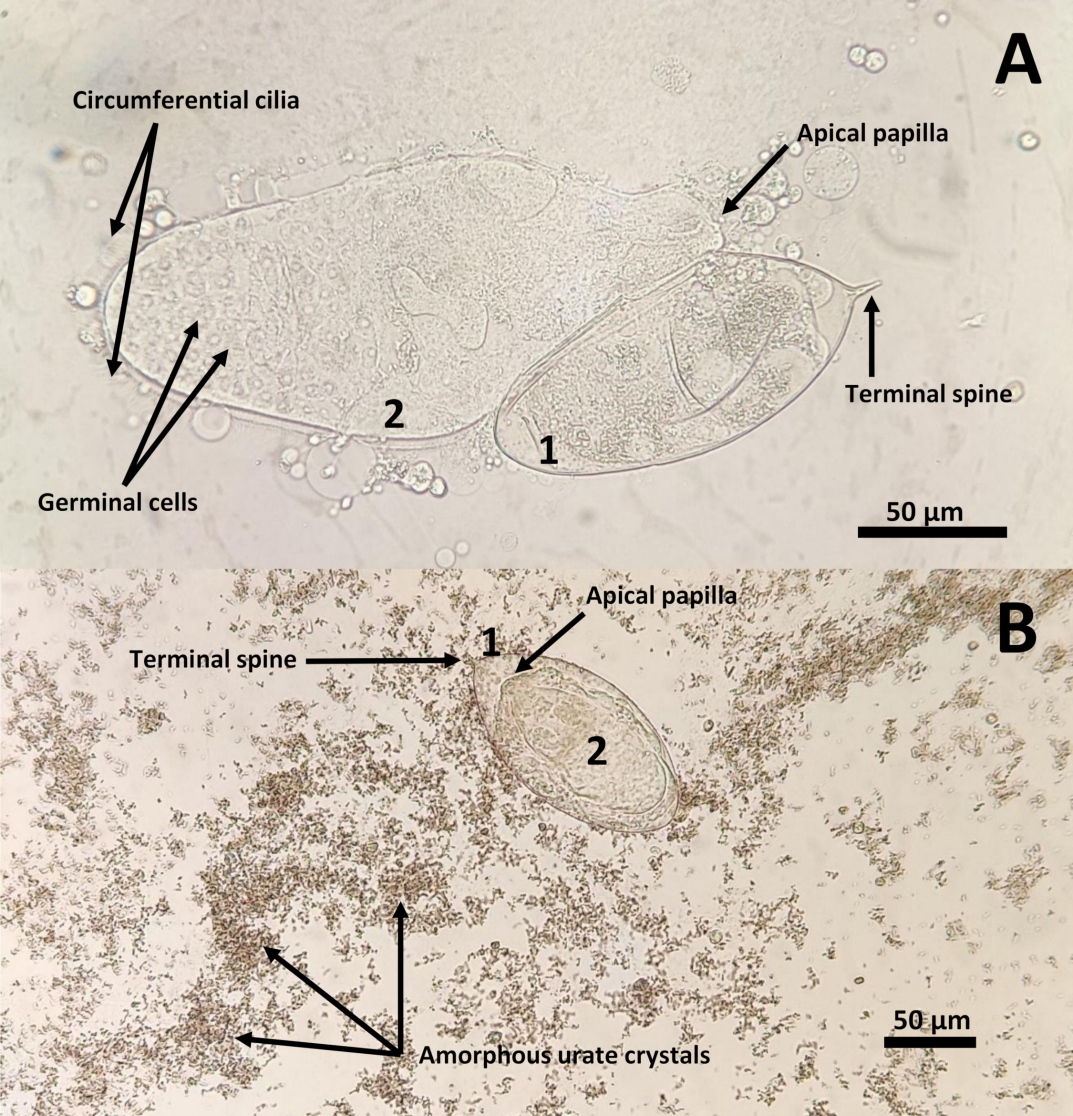
*S. haematobium* miracidium hatching (**A**) and egg (**B**). Panel A (magnification, 400×) shows both egg (1) and free miracidium (2), including internal structures of the miracidium, such as circumferential cilia, apical papilla, and germinal cells. Panel B (magnification, 200×) shows an intact egg (1), with the miracidium inside (2), surrounded by amorphous urate crystals. In both panels, the characteristic terminal spine is apparent. A video of an *S. haematobium* miracidium hatching is viewable at https://youtu.be/AiKnkXMUMak.

Parasitological study revealed both eggs and miracidia of *Schistosoma haematobium* in the patient’s urine, with some hatching eggs and releasing miracidia (Fig. 2). Before starting praziquantel treatment, a complete parasitological study was performed, (including malaria, filaria, and geohelminths). Also, stool PCR detected *Strongyloides stercoralis*, a widespread helminth responsible for strongyloidiasis, with clinical presentation ranging from asymptomatic (as observed in this patient) to fatal disseminated disease, thus the patient was also given ivermectin.

A month later, hematuria had ceased, the urine ova and parasite exam was negative, and *S. stercoralis* was not detected from stool, with the patient staying asymptomatic.

Human schistosomiasis is one of the most prevalent neglected parasitic diseases worldwide, with more than 200 million infected individuals from 78 different countries and more than 700 million at risk living in endemic areas (1)*,* with *S. haematobium* being the most prevalent species in sub-Saharan Africa (2).

One of the typical analytical abnormalities of helminth infections is eosinophilia although is not always present (3), as was observed in this patient.

In the adult population, urinary schistosomiasis is mostly asymptomatic, but it can lead to fertility impairment or the development of bladder cancer caused by squamous cells if left undiagnosed and untreated (4).

Certain snail species play an important role in the *Schistosoma* infective life cycle, serving as the intermediate host. One of these snail species, *Bulinus truncatus,* has been reported in southern Europe, leading to some autochthonous genitourinary schistosomiasis cases, such as the outbreak reported in Corsica, which was caused by both human *S. haematobium*, cattle *Schistosoma bovis*, and the hybrid species (5). Additionally, in south Spain, some autochthonous cases have been reported (6), but no molecular characterization of *Schistosoma* species was made.

Urinary schistosomiasis constitutes a widespread parasitic infection in sub-Saharan Africa, with severe consequences if undiagnosed and untreated. In addition, the increasing international human mobility has led to some autochthonous cases in non-endemic areas. *S. haematobium* screening programs in migrants can contribute to their early diagnosis and treatment, improving migrants’ health and avoiding disease spread to non-endemic areas.
